# Weaning Age Affects the Development of the Ruminal Bacterial and Archaeal Community in Hu Lambs During Early Life

**DOI:** 10.3389/fmicb.2021.636865

**Published:** 2021-03-23

**Authors:** Huiling Mao, Yanfang Zhang, Yan Yun, Wenwen Ji, Zhao Jin, Chong Wang, Zhongtang Yu

**Affiliations:** ^1^College of Animal Science and Technology, College of Veterinary Medicine, Zhejiang A & F University, Hangzhou, China; ^2^Department of Animal Sciences, The Ohio State University, Columbus, OH, United States

**Keywords:** weaning age, postweaning, lamb, rumen, bacteria, archaea, weaning stress

## Abstract

Weaning plays an important role in many animal processes, including the development of the rumen microbiota in ruminants. Attaining a better understanding of the development of the rumen microbial community at different weaning stages can aid the identification of the optimal weaning age. We investigated the effects of weaning age on ruminal bacterial and archaeal communities in Hu lambs. Thirty male Hu lambs were randomly assigned to two weaning-age groups: a group weaned at 30 days of age (W30) and a group weaned at 45 days of age (W45), with each group having five replicate pens. On the weaning day (day 30 for W30 and day 45 for W45) and at 5 days postweaning [day 35 for W30 (PW30) and day 50 for W45 (PW45)], one lamb from each replicate was randomly selected and sacrificed. Rumen contents were collected to examine the ruminal microbiota. Compared to W30, PW30 had a decreased relative abundance of *Bacteroidetes*. At genus level, the extended milk replacer feeding (W45 vs. W30) increased the relative abundance of *Ruminococcus* while decreased that of *Prevotella* and *Dialister*. Compared to W30, PW30 exhibited decreased relative abundances of *Prevotella*, *Dialister* and *Bacteroides* but an increased unclassified *Coriobacteriaceae*. No significant difference was noted in the detected archaeal taxa among the animals. The function “biosynthesis of secondary metabolites” was less predominant in PW30 than in W30, whereas the opposite held true for “metabolism of cofactors and vitamins.” Some bacterial genera were significantly correlated with rumen volatile fatty acid (VFA) concentration or other animal measures, including negative correlations between ruminal VFA concentration and unclassified *Mogibacteriaceae* and unclassified *Veillonellaceae*; positive correlations of ruminal papillae length with *Fibrobacter* and *unclassified Lachnospiraceae*, but negative correlations with *Mitsuokella* and *Succiniclasticum*; and negative correlations between plasma D-lactate concentration and *Prevotella*, unclassified *Paraprevotellaceae*, and *Desulfovibrio*. Our results revealed that the ruminal bacterial community underwent larger changes over time in lambs weaned at 30 days of age than in lambs weaned half a month later. Thus, extending milk replacer feeding to 45 days weaning was recommended from the perspective of the rumen microbial community in the Hu lamb industry.

## Introduction

Early weaning of production animals has many merits; for example, it enhances growth, feedlot performance (Myers et al., 1999a) and carcass quality (Myers et al., 1999b) and shortens reproduction cycles in dams (Thu and Trach, 2012). However, early weaning is a potent stressor that increases morbidity and mortality in young animals, including lambs (Li et al., 2018). The extreme dietary shift imposed by weaning can influence the severity of production losses through the weaning transition (Meale et al., 2016; Mao et al., 2019a). To reduce weaning stress, a weaning strategy is required in which young animals are weaned when they reach solid food and milk or milk replacer allowances are gradually decreased.

During and immediately after birth, the digestive tract of lambs is rapidly colonized by microbiota. Many factors influence microbial colonization, such as the dam’s vaginal microbiome and the types of microbes in the surrounding environment (Meale et al., 2016). Some researchers have found that the rumen microbiome shifts with host age (Jami et al., 2013; Rey et al., 2013). Some studies have investigated the transition of the rumen microbiome induced by weaning (Meale et al., 2017; Li C. et al., 2020). Wang et al. (2016) reported that the influence of weaning on ruminal microflora probably came from the stress of physiology and psychology. Li Y. et al. (2020) have made a research on the dynamics of young forest musk deer intestinal microbiota during the weaning transition, and they believed that weaning stress may affect the composition of the intestinal microbiota. However, the stress of weaning at different ages on the rumen microbiome remains poorly understood. In this study, the effect of weaning age (two ages) on the ruminal microbial communities of Hu lambs was investigated, and differences between postweaning intervals at each weaning age were evaluated. The aim was to identify an appropriate weaning age for lambs from the perspective of the rumen microbial community.

## Materials and Methods

### Study Animals and Sample Collection

As part of a previous study (Mao et al., 2019b), rumen samples were collected from Hu lambs. Briefly, 30 healthy male Hu lambs (3.60 ± 0.37 kg) at 5 days of age were selected, maintained at a breeding farm and fed milk replacer (MR). The lambs were randomly assigned to two weaning-age groups: one group weaned at 30 days of age (W30) and one group weaned at 45 days of age (W45). Each group has 15 lambs, and three lambs with similar body weight within the same group were placed in one pen. Individual pens were considered as experimental replicates (*n* = 5). On the weaning day (day 30 for the W30 group and day 45 for the W45 group) and at five days postweaning [day 35 for the W30 group (PW30) and day 50 for the W45 group (PW45)], one lamb of each replicate per group was randomly selected and sacrificed (*n* = 5). All the lambs were also offered, *ad libitum*, starter pellets and hay of Chinese wild rye from day 10 (Supplementary Table 1). Body weight were recorded for 2 consecutive days every 10 days before morning feeding and average daily gain was calculated. Before slaughter, blood samples were collected, and plasma was prepared to determine the concentration of D-lactate. Immediately after slaughter, rumen content samples were collected and stored at −80°C for subsequent microbial DNA extraction, and rumen tissues were sampled to measure ruminal papillae length. Additional procedures are as described in Mao et al. (2019b), with the exception that in the present study, data collected on the day of animal sacrifice were used for bacterial and archaeal analysis.

### DNA Extraction and Data Processing

The cetyltrimethyl ammonium bromide method as described by Gagen et al. (2010) was used to extract the total DNA of the rumen content samples. The hypervariable V3-V4 region of the bacterial and archaeal 16S rRNA gene was amplified using primers 341F (5′-ACTCCTACGGGRSGCAGCAG-3′) and 806F (5′-GGACTACVVGGGTATCTAATC-3′) (Wang and Qian, 2009). Each primer had six base barcodes to identify samples. The amplicon libraries were pooled at an equimolar ratio and sequenced using the 2 × 250 paired-end protocol on an Illumina HiSeq platform (Realbio Genomics Institute, Shanghai, China).

Microbiome bioinformatics were performed with QIIME 2 (Bolyen et al., 2018). The paired-end sequencing reads were assembled using the PANDAseq assembler (Masella et al., 2012). Operational taxonomic units (OTUs) were clustered at a 97% identity threshold, and taxa were assigned using the core set in the Greengenes 16S reference database (13_8 version) (McDonald et al., 2012). Alpha diversity measurements of the ruminal bacterial and archaeal communities including the observed_OTUs, Shannon index (Shannon and Weaver, 1949), Faith’s phylogenetic diversity (Faith, 1992), and evenness calculated by QIIME 2. The overall dissimilarity of the microbial community between weaning ages was evaluated by principal coordinates analysis (PCoA) based on unweighted UniFrac distances (Lozupone and Knight, 2005). The significance of differences between groups was tested by analysis of similarity (ANOSIM). A significant difference was declared at 0.5 < *R* < 0.75 with *P* < 0.05, whereas a trend was declared at 0.3 < *R* < 0.5 with *P* < 0.05, and no difference was declared at *R* < 0.3. Data on plasma D-lactate concentration, rumen papillae length and width, and ruminal total VFA concentrations were obtained from our previous study (Mao et al., 2019b) and analyzed for correlations with bacterial taxa. Based on comparison of the 16S rRNA gene sequences against a Green genes reference taxonomy (Green genes 13.8), the functional capabilities of the rumen microbiota were predicted by using PICRUSt (Phylogenetic Investigation of Communities by Reconstruction of Unobserved States) (Langille et al., 2013). Briefly, after the abundance of each OTU was normalized to marker gene copy number, the KEGG database was used to predict the functions.

### Statistical Analysis

The alpha diversity indices (observed numbers of OTUs, Shannon index, Faith’s phylogenetic diversity, and evenness) of bacteria and archaea, and phenotypic data (plasma D-lactate concentration, ruminal papillae length and width, and ruminal VFA concentration) were analyzed by one-way ANOVA using the general linear model procedure of SAS (SAS Inst. Inc., Cary, NC, United States). Statistical significance was set at *P* ≤ 0.05. For the relative abundances of rumen bacteria, all the taxa analyzed in the present study were identified in at least three lambs at each group. It meant that only the bacteria that were observed in at least 60% of samples were considered in the relative abundance analysis. To show the changes in bacterial relative abundances, bacterial data were subjected to LEfSe analysis (Segata et al., 2011). A significant change was observed with a LDA (Linear Discriminant Analysis) score > 2.0 calculated by LEfSe. Spearman’s rank correlations between the relative abundances of major (ruminal bacterial and archaeal taxa (i.e., taxa with relative abundances > 0.5%) and plasma D-lactate, ruminal papillae length and width, and ruminal VFA concentration were analyzed using the PROC CORR procedure of SAS. The correlation coefficients were plotted using GraphPad Prism 7.

## Results

### Bacterial Community Composition

At least 43,063 sequences were obtained from each sample, and 575 operational taxonomic units (OTUs, 144 ± 28 OTUs per sample) defined based on 97% similarity were detected. Good’s coverage for each sample was greater than 99%, indicating sufficient sequencing depth to detect most of the rumen bacteria of the Hu lambs in this study. As shown in Table 1, extending MR feeding for 15 days increased the number of observed OTUs and Faith’s phylogenetic diversity (*P* < 0.01), but there was no significant difference in any of the alpha diversity indices between weaning and postweaning (*P* > 0.05).

**TABLE 1:** Alpha diversity index values of ruminal bacteria and archaea in different groups.

Domain	Indices	Treatments				SEM	*P*-value
		
		W30	PW30	W45	PW45		
Bacteria	Observed OTUs	123^b^	121^b^	164^a^	167^a^	10.0	<0.01
	Shannon	5.00^ab^	4.38^b^	4.92^ab^	5.30^a^	0.260	0.15
	PD index^1^	9.81^b^	9.35^b^	11.9^a^	12.3^a^	0.51	<0.01
	Evenness	0.72	0.63	0.67	0.72	0.034	0.25
Archaea	Observed OTUs	3^b^	6^a^	6^a^	8^a^	0.74	<0.01
	Shannon	0.64^b^	1.22^b^	1.40^ab^	2.19^a^	0.263	0.01
	PD index^1^	0.11^b^	0.20^a^	0.21^a^	0.23^a^	0.017	<0.01
	Evenness	0.33^b^	0.49^ab^	0.54^ab^	0.73^a^	0.087	0.05

*^1^Faith’s phylogenetic diversity.*

*^*a,b*^Means within a row with different superscripts differ significantly (*P* < 0.05).*

**FIGURE 1:**
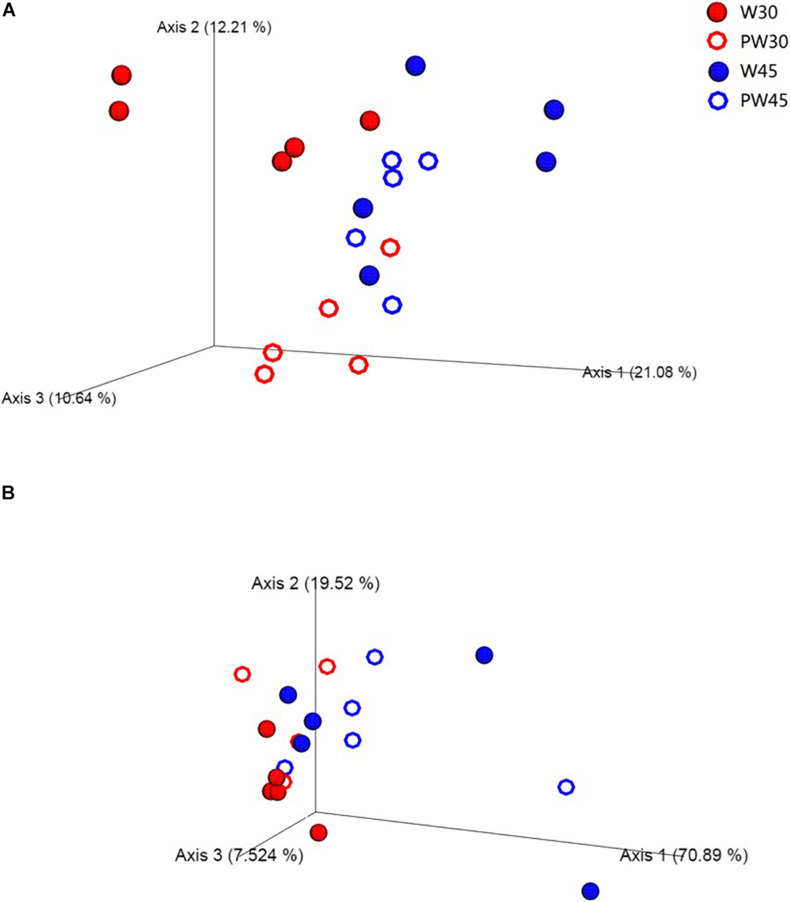
Principal coordinates analysis (PCoA) of bacterial **(A)** and archaeal **(B)** communities in the rumen based on unweighted UniFrac distances.

The PCoA showed that extending the period of MR feeding significantly altered the rumen bacterial composition (Figure 1A). ANOSIM revealed significant differences in bacterial community composition between treatments W30 and PW30 (*R* = 0.594, *P* = 0.032), W30 and W45 (*R* = 0.521, *P* = 0.032), and PW30 and PW45 (*R* = 0.646, *P* = 0.027). No significant difference was found between treatments W45 and PW45 (*R* = 0, *P* = 0.493).

**FIGURE 2:**
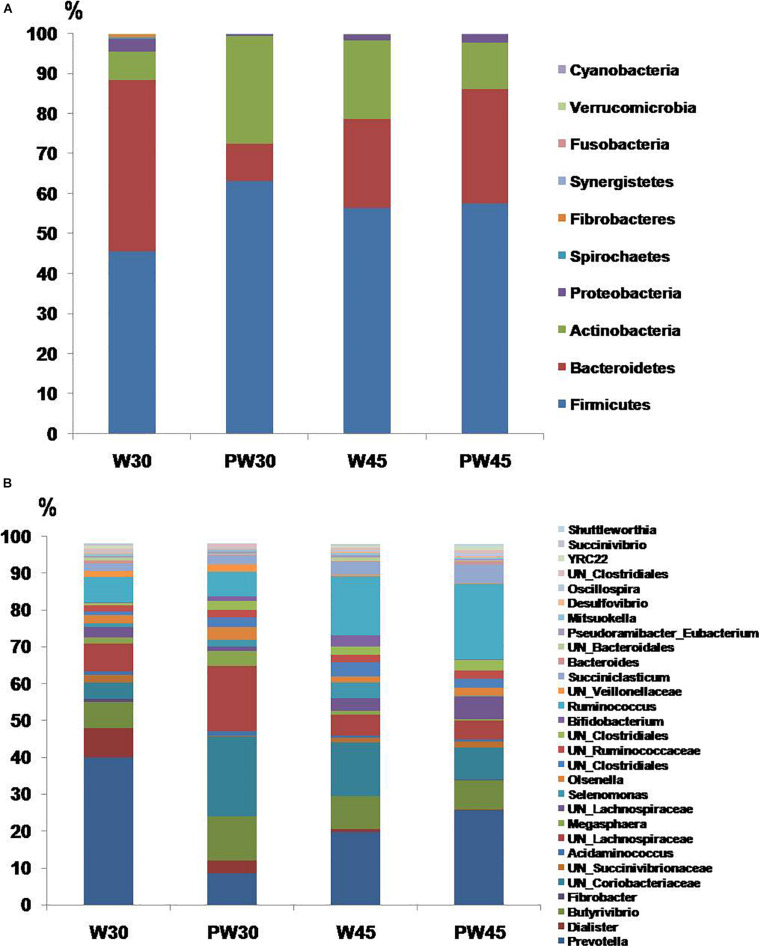
Ruminal bacterial phyla **(A)** and genera **(B)** in the four groups. Only those genera with relative abundances of >0.5% are shown.

**FIGURE 3:**
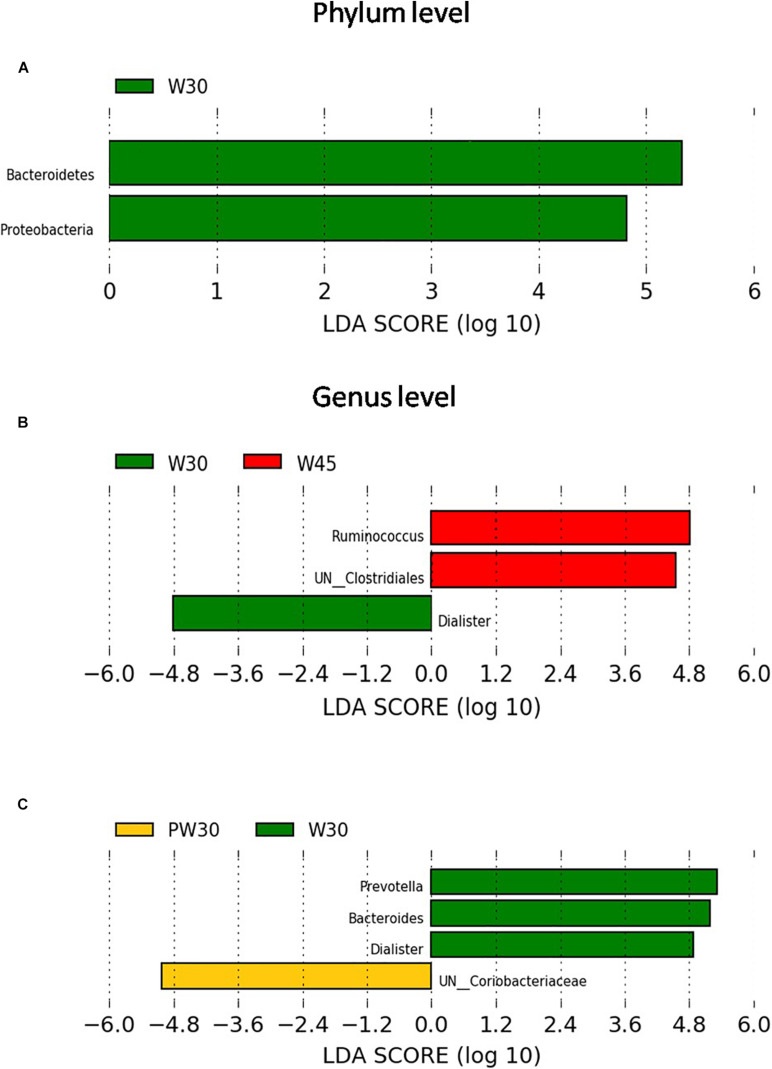
Phylum level changes between W30 and PW30 **(A)**, no differentially abundant features were found neither between W30 and W45 nor between W45 and PW45. Genus level changes between W30 and W45 **(B)**, and between W30 and PW30 **(C)**, no differentially abundant features were found between W45 and PW45.

Ten bacterial phyla were detected. *Firmicutes, Bacteroidetes, Actinobacteria*, and *Proteobacteria* were the four predominant phyla, representing 55.7, 25.7, 16.3, and 1.91% of the total sequences, respectively (Figure 2A). The relative abundance of *Bacteroidetes and Proteobacteria* were increased in W30 compared with PW30 (Figure 3A). However, there were no significant differences in the relative abundances of any of the phyla between W45 and PW45. A total of 108 bacterial genera were detected, of which 29 had relative abundances greater than 0.5% (Figure 2B). With the extension of MR feeding (W45 vs. W30; Figure 3B), the relative abundances of *Prevotella* and *Dialister* decreased, whereas the abundance of *Ruminococcus* increased. Among the lambs weaned at 30 days (Figure 3C), the abundances of *Prevotella*, *Dialister* and *Bacteroides* decreased from weaning to 5 days postweaning, whereas *UN_Coriobacteriaceae* abundance increased. However, there was no differentially abundant features were found between weaning and postweaning among the lambs weaned at 45 days.

### Archaeal Community Composition

Following denoising and chimera checking, an average of 4,686 archaeal sequences per rumen sample were obtained, with an average of six OTUs per sample. Analysis of the archaeal 16S rRNA gene sequences revealed that weaning age had a significant effect (*P* < 0.01) on the number of observed OTUs and Faith’s phylogenetic diversity (Table 1) and that both of these alpha diversity indexes increased from weaning to postweaning (*P* < 0.01) among the lambs weaned at 30 days but not among those weaned at 45 days (Table 1). A PCoA of overall archaeal community diversity was performed to examine differences among the four treatments (Figure 1B). ANOSIM revealed significant differences in archaeal community composition between treatments W30 and PW30 (*R* = 0.625, *P* = 0.036) and treatments W30 and W45 (*R* = 0.562, *P* = 0.038). However, there was no significant difference between treatments in any other comparisons (*P* > 0.05).

Only one phylum, *Euryarchaeota*, was identified in all the rumen samples. We detected two genera of *Euryarchaeota* in all of the treatments, with *Methanobrevibacter* being much more predominant (92.3–97%) than *Methanosphaera* (3–7.7%). There was no significant difference in the relative abundance of either of these two genera among the four treatment groups (*P* > 0.05).

### Predicted Rumen Microbial Functions

**FIGURE 4:**
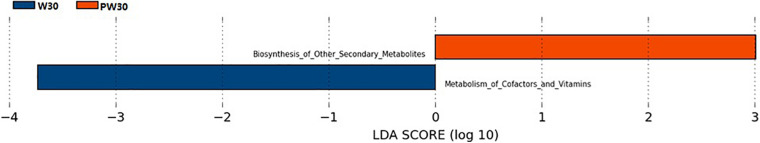
Differences in predicted metagenomic functions of rumen bacteria between the W30 and PW30 groups of lambs. No functional feature was found to differ between W45 and PW45 lambs by LEfSe.

The functional features predicted from the 16S rRNA sequences did not differ (P > 0.05) in relative abundance between the two weaning ages (W30 vs. W45) or between weaning and 5 days postweaning among the lambs weaned at 45 days (W45 vs. PW45). However, the relative abundance of sequences associated with the function “biosynthesis of secondary metabolites” significantly increased (*P* < 0.05) from weaning to postweaning among the lambs weaned at 30 days (W30 vs. PW30) (Figure 4).

### Relationships Between Bacterial Taxa and Phenotypic Variables

**FIGURE 5:**
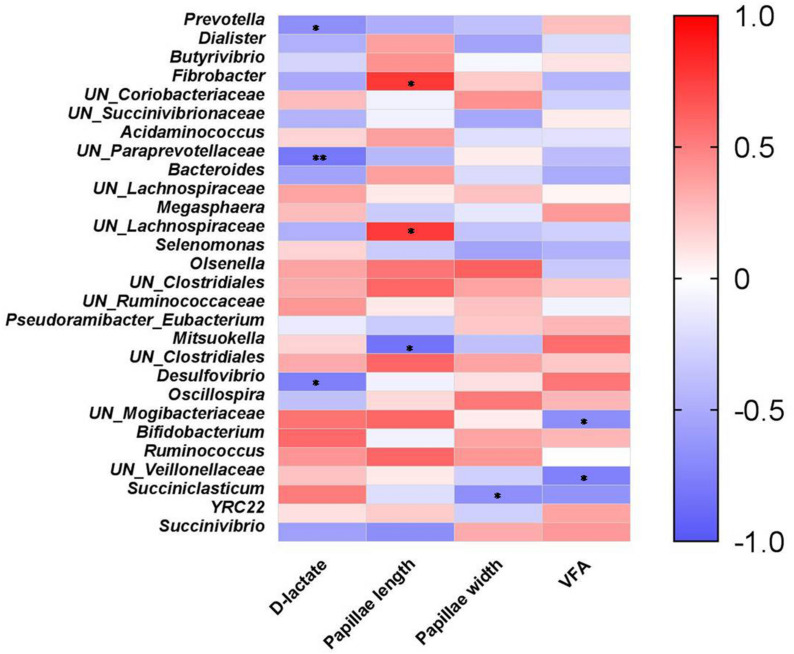
Spearman’s correlation coefficients for individual major rumen bacterial genera (each with >0.5% relative abundance) and phenotypic variables. Color represents the correlation coefficient, with red representing a positive correlation and blue denoting a negative correlation. *P < 0.05 and **P < 0.01.

The plasma D-lactate, VFA, ruminal papillae length and width of W30 and W45 were obtained from our previous study (Mao et al., 2019b), and re-analyzed with data of 20 lambs for bacteria analysis (Table 2). Correlation analysis showed that plasma D-lactate was negatively correlated (Figure 5) with the relative abundances of *Prevotella* (*P* < 0.05), *unclassified Paraprevotellaceae* (*P* < 0.01) and *Desulfovibrio* (*P* < 0.05). Ruminal papillae length was positively correlated with the relative abundances of *Fibrobacter* (*P* < 0.05) and unclassified *Lachnospiraceae* (*P* < 0.05) but negatively correlated with the relative abundance of *Mitsuokella* (*P* < 0.05). Ruminal papillae width was negatively correlated with only the relative abundance of *Succiniclasticum* (*P* < 0.05). Ruminal total VFA concentration was negatively correlated with the relative abundances of unclassified genera within *Mogibacteriaceae* (*P* < 0.05) and *Veillonellaceae* (*P* < 0.05).

**TABLE 2:** Phenotypic variables of Hu lambs of different groups.

Items	Treatments				SEM	*P*-value
	
	W30	PW30	W45	PW45		
D-lactate, ng/mL	1024^ab^	1081^a^	950^*c*^	976^*bc*^	20.4	<0.01
Rumen papillae						
Length, μm	581^b^	814^ab^	985^ab^	1307^a^	153	0.08
Width, μm	323	362	369	383	36.6	0.26
Total VFA, mg/ml	1.58^ab^	2.38^a^	2.50^a^	1.08^b^	0.314	0.05

*^*a*,*b*,*c*^Means within a row with different superscripts differ significantly (*P* < 0.05).*

## Discussion

Understanding the impacts of weaning age on animal growth, rumen tissue structure, and the structure and functionality of the rumen microbiome during early life and the changes that occur postweaning can aid the identification of an appropriate weaning age for achieving optimal health and growth performance throughout adulthood. In this study, we characterized the rumen microbiota at two different weaning ages (30 vs. 45 days) and the changes that occur postweaning (5 d) in the diversity, composition and predicted functional features of the rumen bacterial and archaeal communities. The lambs in the two weaning-age groups received the same diet and exhibited similar levels of feed intake (Mao et al., 2019b), allowing us to determine the effects of weaning age on the aforementioned measures. Additionally, we investigated whether weaning stress influenced microbial colonization in the rumen, information that may aid the selection of an appropriate weaning age.

The gastrointestinal tract of most newborn animals is thought to be sterile, but at birth, microbes from the surrounding environment and the dam rapidly colonize the rumen (Yáñez-Ruiz et al., 2015). Jami et al. (2013) reported that some rumen bacteria essential for mature rumen function were present as early as 1 day after birth, and Li et al. (2012) found that all major types of rumen bacteria could be detected in the rumen of 14-day-old calves. Guzman et al. (2015) suggested that inoculation occurs even before birth, noting that members of typical functional bacteria have been found in the rumen of calves less than 20 min after birth. However, there was no significant difference in abundance of any of the top four bacterial phyla between the two weaning ages (W30 and W45) in this study. This finding suggests that the rumen microbiota might have largely achieved stability by the age of 30 days while lambs were consuming the same diet. This interpretation is in general agreement with Yáñez-Ruiz et al. (2015) finding that the rumen microbiota no longer exhibited clear temporal changes at the phylum level beyond 15 days of age. Furthermore, Meale et al. (2016) suggested that the developing gut (preweaning) contains the same dominant phyla as the more mature gut, although the relative abundances vary with developmental stage. In the present study, the relative abundance of *Bacteroidetes* decreased from 0 to 5 days postweaning among the lambs weaned early (at day 30). Interestingly, none of the major bacterial phyla exhibited significant changes in relative abundance from 0 to 5 days postweaning among the lambs weaned at 45 days of age. This finding suggests that early weaning stress could have a negative effect on rumen development. *Bacteroidetes* plays a role in the normal development of the digestive tract, affecting traits such as the growth and volume increase of the rumen (Thomas et al., 2011; Lin et al., 2018).

Seven genera exhibited significant differences in relative abundance among treatments. While it did not mean these seven microbial genera have the most important functions in the complex rumen environment. Many microbes share the same metabolic pathways in the rumen; thus, a significant change in microbial community composition may not entail a shift in function (Yang et al., 2018). Consistent with this observation, Li et al. (2012) found that all of the functional classes in the rumen were similar between 14-day-old and 42-day-oldcalves. In this study, extension of the MR feeding period decreased the abundances of *Prevotella* and *Dialister* and increased the abundance of *Ruminococcus*. *Prevotella* strains are highly amylolytic and proteolytic (Matsui et al., 2000; Xu and Gordon, 2003). Members of *Dialister* have been reported to play roles in altering the buffering capacity of the rumen and fluid turnover (Myer et al., 2015). The members of *Ruminococcus* are cellulolytic, fiber-degrading bacteria (Ezaki, 2015) and can produce all organic acids. Zhong et al. (2019) found that increasing the relative abundance of *Ruminococcaceae* increased butyrate production during the transition from weaning to postweaning, which stimulating jejunal adaption toward gut health in piglets. The results of the present study suggested that extending the duration of MR feeding induces microbial changes and potentially affects rumen digestion. From weaning to postweaning, the abundances of *Prevotella*, *Dialister*, *and Bacteroides* decreased, and the abundance of unclassified Coriobacteriaceae increased among lambs weaned at 30 days of age, whereas no abundance differences between weaning and postweaning were observed among lambs weaned at day 45. These findings suggest that early weaning stress could change the microbial composition, and Bailey et al. (2011) also believed that the relative abundance of bacteria in the genus *Bacteroides* decrease under the influence of stressors. Weaning stress may be an reason for the decrease in *Bacteroides* after weaning (Li Y. et al., 2020). Additionally, extending the period of MR feeding seems to increase rumen microbial diversity. A high level of diversity could provide “functional redundancy” that makes the ecosystem more resilient to environmental stressors and more stable (Konopka, 2009). In our previous study (Mao et al., 2017), rumen microbial changes in response to weaning were observed; however, only some known cellulolytic bacteria were quantified, and postweaning changes were not investigated. Therefore, the present study provides a more comprehensive assessment of how weaning affects rumen microbial colonization, taking weaning stress into consideration.

In the present study, only *Euryarchaeota* phylum was identified in all the rumen samples. This finding was similar to Kumar et al. (2015), who reported *Euryarchaeota* was the dominant archaea phylum in the rumen microbiome. Moreover, archaeal communities were concluded to less diverse (3–4% of the rumen microbiome) and less variable than other microbial domains (Jeyanathan et al., 2011), which might be due to the establishment of stable methanogenic communities in the rumen at a very early age (Su et al., 2014).

Based on the PICRUSt analysis of the rumen microbiome, we speculate that weaning stress of W45 lambs was lower than that of W30 lambs. However, despite a lower abundance of the genus *Prevotella* in the lambs weaned at 30 days than in those weaned at 45 days, no significant differences in amino acid metabolism were observed between these groups. This finding may have been due to the establishment of the functional maturity of the rumen prior to microbial maturity. Meale et al. (2016) found that among the 10.75% of OTUs assigned to a KO category, none were associated with significant changes in carbohydrate metabolism. However, the predictions of PICRUSt are based on known microbial functions, and many of the functions of unclassified bacteria may be over- or underestimated (Yang et al., 2018). Wilkinson et al. (2018) indicated that the functional profiles predicted by CowPI better match estimates for both the meta-genomic and transcriptomic datasets than PICRUSt. On the other hand, metagenomic and metabolomic analyses might be used to analyze the functions of the rumen microbiome to better understand the effects of changes of bacterial taxa on rumen function, functional inferences from 16S data should not replace metagenomic and metatranscriptomic approaches. Therefore, we could improve the function prediction in the future study.

The VFAs in the rumen stimulate rumen epithelial metabolism (Baldwin and McLeod, 2000), and butyrate has been shown to be the most stimulatory (Górka et al., 2011). Butyrate promotes cell proliferation and inhibits cell apoptosis, leading to the functional maturation of intestinal epithelial cells (Hamer et al., 2008; Zhong et al., 2019). The genus *Butyrivibrio* represents a major group of butyrate producers in the rumen (Bryant, 1986). In the present study, the abundance of this genus was positively correlated with papillae length in the rumen. This result is consistent with that of Yang et al. (2018). We observed a positive correlation between *Succinivibrio* abundance and rumen VFA concentration, which corroborates the finding of Rey et al. (2013), who found a similar correlation in the rumen of 3- to 12-day-old calves.

## Conclusion

In conclusion, this study provides new insights into the colonization of the weaning-lamb rumen and associations within the rumen microbiota. The results suggest that extending the period of MR feeding could influence the microbial composition and potentially benefit rumen development and mitigate the negative effect of weaning stress. Weaning at the age of 45 days may be recommended from the perspective of the rumen microbial community.

## Data Availability Statement

The datasets generated for this study can be found in the NCBI sequence read archive under BioProject PRJNA683734.

## Ethics Statement

The animal study was reviewed and approved by Animal Use and Care Committee, Zhejiang A & F University (Hangzhou, China).

## Author Contributions

HM and CW: conceptualization and methodology. HM and YZ: investigation and data collection. YY, WJ, and ZJ: sample analysis. CW: formal statistical analysis. HM: writing-original draft preparation, project administration, and funding acquisition. ZY: writing-review and editing. All authors contributed to the article and approved the submitted version.

## Conflict of Interest

The authors declare that the research was conducted in the absence of any commercial or financial relationships that could be construed as a potential conflict of interest.

## Abbreviations

ANOSIM: analysis of similarity
ANOVA: analysis of variance
DNA: deoxyribonucleic acid
KO: KEGG orthology
LEfSe: linear discriminate analysis effect size
MR: milk replacer
OTU: operational taxonomic unit
PCoA: principal coordinate analysis
PICRUSt: phylogenetic investigation of communities by reconstruction of unobserved states
VFA: volatile fatty acid.
