# Supplementary figures and images for: Weaning Age Affects the Development of the Ruminal Bacterial and Archaeal Community in Hu Lambs During Early Life
Source: Front Microbiol. 2021 Mar 23;12:636865. doi: 10.3389/fmicb.2021.636865 (PMC8021712; doi:10.3389/fmicb.2021.636865)

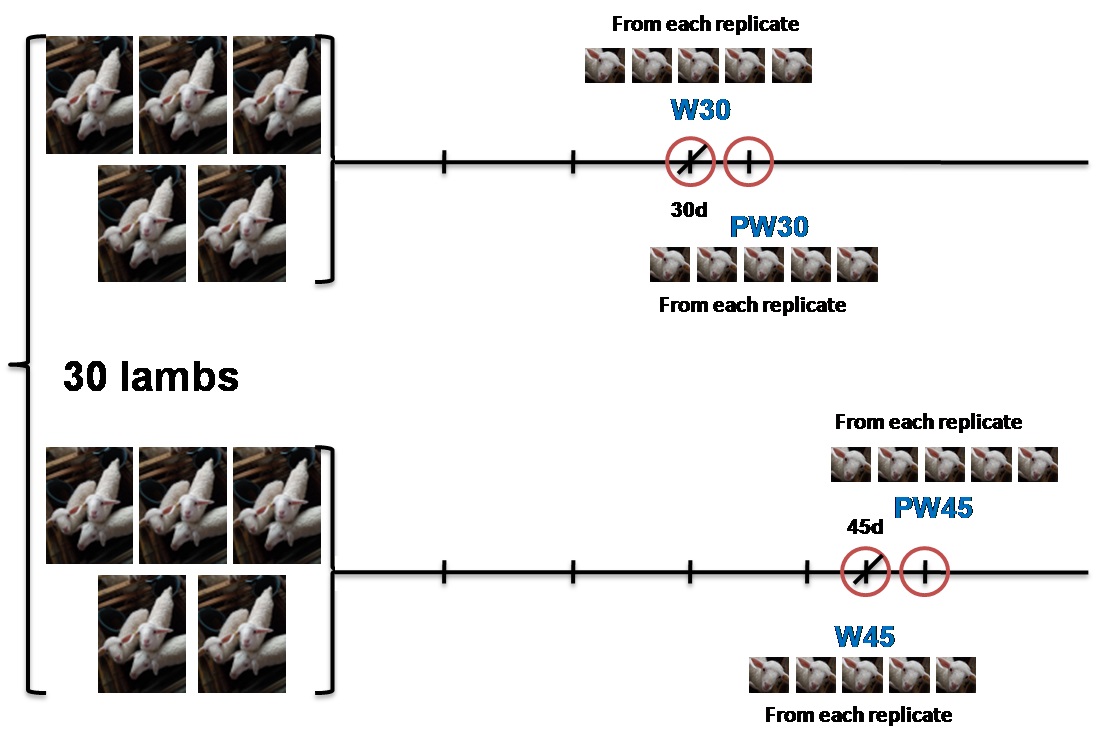

Supplement: Supplementary file 1 [file Image_1.JPEG]
