# Supplementary material for: Weaning Age Affects the Development of the Ruminal Bacterial and Archaeal Community in Hu Lambs During Early Life
Source: Front Microbiol. 2021 Mar 23;12:636865. doi: 10.3389/fmicb.2021.636865 (PMC8021712; doi:10.3389/fmicb.2021.636865)
Supplement: Supplementary file 2 [file Table_1.DOCX]

**Table S1.** Chemical composition of the feeds (g/kg, DM basis)

| Items^a^ | Milk replacer | Starter pellets^b^ | [Chinese](javascript:void(0);) [wild rye](javascript:void(0);) hay |
| --- | --- | --- | --- |
| DM | 945 | 914 | 929 |
| Crude protein | 184 | 174 | 84.9 |
| NDF | 36 | 169 | 659 |
| ADF | 9.0 | 73 | 384 |
| Calcium | 7.2 | 12.1 | 2.4 |
| Phosphorus | 6.3 | 5.9 | 1.0 |
| Ash | 41 | 73 | 60 |

^a^ DM = dry matter; NDF = neutral detergent fibre, assayed without the addition of sodium sulfite and amylase; ADF = acid detergent fibre

^b^ The starter pellets consisted of 58.5g/100g corn, 10g/100g wheat meal, 27g/100g soybean meal, 1.1g/100g CaHCO_3_, 1.4g/100g limestone, 1g/100g NaCl, and 1g/100g vitamin-mineral premix.
